# Cellulose–Chitosan Functional Biocomposites

**DOI:** 10.3390/polym15020425

**Published:** 2023-01-13

**Authors:** Simona Strnad, Lidija Fras Zemljič

**Affiliations:** Faculty of Mechanical Engineering, University of Maribor, Smetanova Ulica 17, 2000 Maribor, Slovenia

**Keywords:** biocomposites, functional materials, cellulose–chitosan, fibres, films, hydrogels, nanofibers

## Abstract

Here, we present a detailed review of recent research and achievements in the field of combining two extremely important polysaccharides; namely, cellulose and chitosan. The most important properties of the two polysaccharides are outlined, giving rise to the interest in their combination. We present various structures and forms of composite materials that have been developed recently. Thus, aerogels, hydrogels, films, foams, membranes, fibres, and nanofibres are discussed, alongside the main techniques for their fabrication, such as coextrusion, co-casting, electrospinning, coating, and adsorption. It is shown that the combination of bacterial cellulose with chitosan has recently gained increasing attention. This is particularly attractive, because both are representative of a biopolymer that is biodegradable and friendly to humans and the environment. The rising standard of living and growing environmental awareness are the driving forces for the development of these materials. In this review, we have shown that the field of combining these two extraordinary polysaccharides is an inexhaustible source of ideas and opportunities for the development of advanced functional materials.

## 1. Introduction

Cellulose and chitin are the most abundant biopolymers on earth. Both are so-called structural polysaccharides, as they build hierarchical structures in plants and animals. The accidental discovery of the synthesis of cellulose nitrate in 1845 [1] and the first successful conversion of insoluble chitin to its soluble derivative in 1859 [2] opened the doors for numerous applications of cellulose and chitosan. With the development of extraction, derivatisation, and solubilisation processes, both polymers have become enormously important in the development of materials and products relevant to virtually all aspects of human life, e.g., food, clothing, health and medical devices, pharmaceuticals, filtering and purification materials, construction materials, etc.

In the 1990s, the number of reports on the successful combination of the two biopolymers increased. The articles in this area reported the production of films from cellulose and chitosan blends [3,4,5]. The consideration of combining the two polymers was based on the fact that both polymers are similar in molecular structure, i.e., they have a highly functionalised, stiff-chain, linear molecular structure, but have different properties that can be combined usefully. Cellulose primarily offers hydrophilicity, structuring and mechanical properties, while chitosan has an electron donor function and antimicrobial properties due to the amino group present. 

The knowledge of the molecular-level interactions between cellulose and chitosan has been crucial for the development of composite materials. The research groups of Holmberg [6] and Laine [7] provided some essential information about such interactions. Based on these theoretical studies, various structures and forms of cellulose–chitosan composites have been developed to date. Films, aerogels, hydrogels, membranes, fibers, etc., have been prepared and studied in detail to utilise all the essential functions of cellulose and chitosan. 

The use of biodegradable polymers or biopolymers is particularly important because they are an excellent alternative to petroleum-based products. There is a great need to develop biopolymer composites to provide environmentally friendly materials and thus reduce the carbon footprint. Moreover, the essential properties of the two biopolymers chitosan and cellulose, such as renewability, sustainability, non-toxicity, biodegradability, and specific functionality, will lead to a new generation of multifunctional composites used for a wide range of applications.

**Figure 1:**
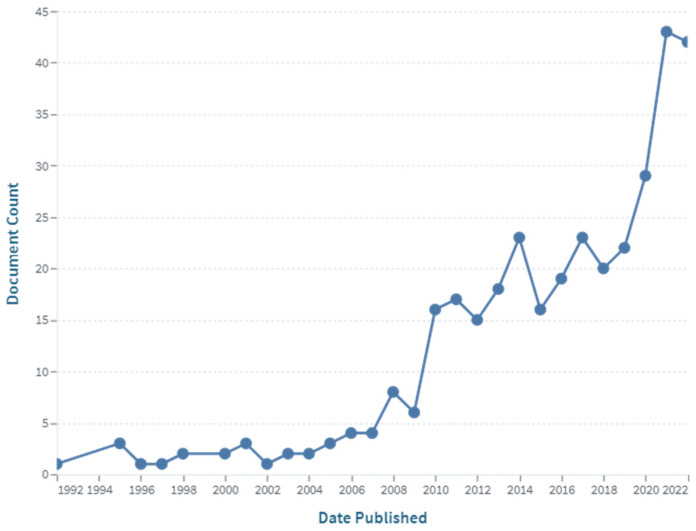
The number of publications with the keyword “cellulose–chitosan” (in the abstract) over time (source: www.lens.org; accessed on 21 December 2022).

**Figure 2:**
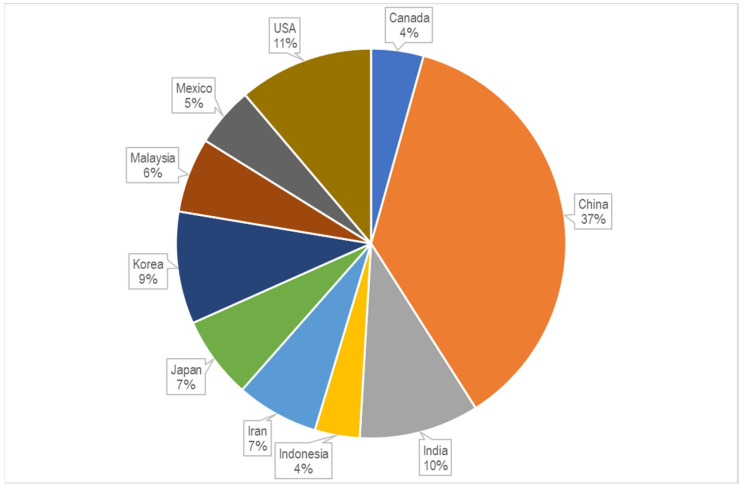
Shares of publications per country or region (source: www.lens.org; accessed on 21 December 2022).

To date, there are 3757 publications including the key words “cellulose” and “chitosan” in their abstracts, but there are only 350 publications with the narrow word combination “cellulose-chitosan” OR “chitosan-cellulose” in their abstracts, and there are only around 50 patent records with the same keyword combination in their titles, with most of them published after 1994. The first two journal articles were published in 1992, and concerned “Characterization of cellulose–chitosan blended films” [3] and “Preparation of cellulose–chitosan polymer blends” [4]. These two articles, written by the same research group, are among the first to report on the combinations and synergies of the two biopolymers with their special properties. It can be seen from Figure 1 that the number of publications on this topic increased significantly after 2011, and again after 2019, confirming the growing interest in the research and development of this type of material. Of the above 204 publications, 51 scientific papers specifically use the keyword combination “cellulose–chitosan composites” in their titles. The first articles of this type were published by Vikhoreva et al. in 2000 [8] and Yang et al. in 2002 [9]. The first group reported the preparation of polysaccharide films with a high degree of swelling from dispersions of powdered cellulose in chitosan solutions, and the second group reported the coating of a filter paper with chitosan to produce a composite membrane that can be used in immunoadsorption with affinity membranes. The authors’ affiliations were Russia, Canada, and China. As can be seen from the chart in Figure 2, most of the publications are from China, the United States, India, and the Republic of Korea.

In this review, we aim to present the scope and complexity of the combination of the two extraordinary biopolymers, cellulose and chitosan, and their importance for the development of special materials and products. The various structures and forms of composites that have been developed and analysed recently are presented, including porous structures such as aerogels, hydrogels, membranes, and foams, as well as films, and, of course, fibres, among which special attention is paid to nanofibers. The coating and fabrication of thin films on various substrates is presented as an important method for the fabrication of composite materials. Special attention is given to bacterial cellulose and various approaches to combining it with chitosan.

Since this is a very extensive and attractive research topic, we had to limit ourselves to blends and composites of unmodified cellulose and chitosan, and their derivatives are mentioned only to illustrate some points. The variety of derivatives of the two biopolymers is another important and broad area of research and development that deserves special treatment and publication.

## 2. Chitosan

**Figure 3:**
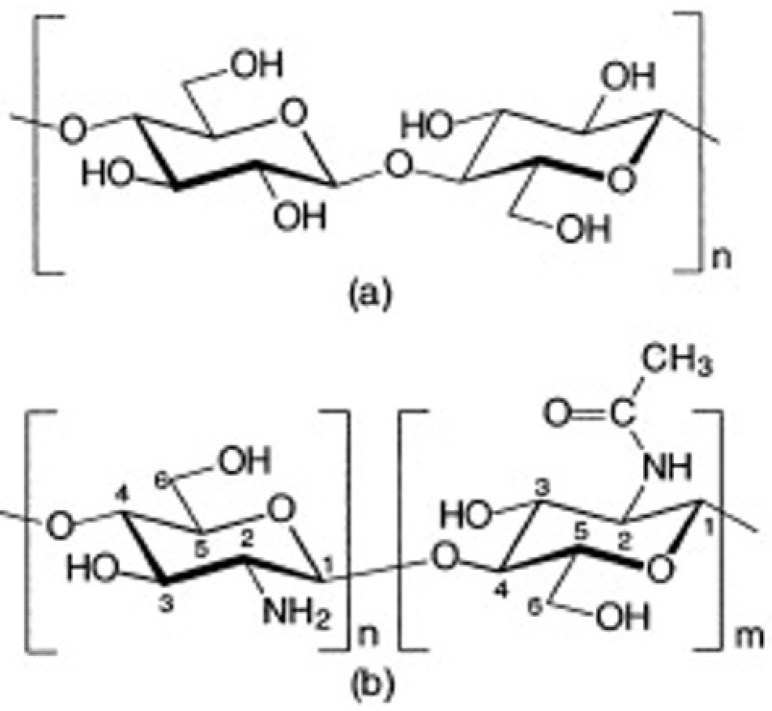
Chemical structure of cellulose (**a**), chitin and chitosan (**b**)—chitin consists mainly of monomers “m” (N-acetyl form), while chitosan, according to the degree of deacetylation, consists mainly of monomers “n” (amine form) [15]. Review of Chitosan and Its Derivatives as Antimicrobial Agents and Their Uses as Textile Chemicals. Lim, Sang-Hoon, Hudson, Samuel M., *Journal of Macromolecular Science*, Part C, 6 January 2003; reprinted with permission of the publisher Taylor & Francis.

Chitosan occurs naturally only in certain fungi [10,11,12]; otherwise, it is a collective name for chitins deacetylated to degrees higher than 50% [13]. It is actually the copolymer of N-acetyl-D-glucosamine and glucosamine (Figure 3), in which the share of glucosamine is usually above 80% [14]. 

**Figure 4:**
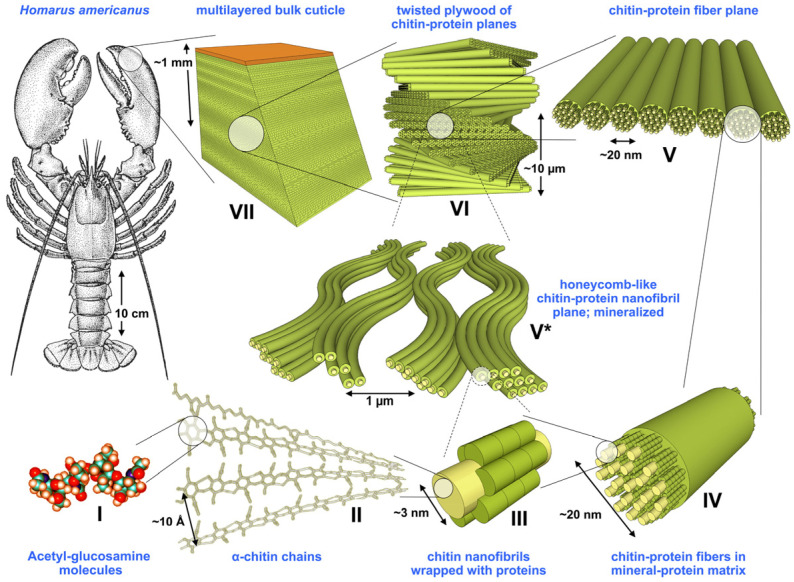
Structural hierarchy in an arthropod cuticle. Levels I to VII represent the general model assumed for the organic matrix in the cuticle; [17]. Reprinted from the *Journal of the Mechanical Behaviour of Biomedical Materials*, 4/2, S. Nikolov, H. Fabritius, M. Petrov, M. Friák, L. Lymperakis, C. Sachs, D. Raabe, J. Neugebauer, Robustness and optimal use of design principles of arthropod exoskeletons studied by ab initio-based multiscale simulations, 129–145, Copyright (2011), with permission from Elsevier.

Chitin (C_8_H_13_O_5_N)_n_ is known to form microfibrillar arrangements in living organisms [16]. Fibrils are usually embedded into a protein matrix forming the typical hierarchical composite structure of skeletons (Figure 4). 

The name “chitine” (from the Greek “chiton”) was first mentioned by Odier in 1823, as he found it in the elytra of insects. Chitin and cellulose have similar molecular structures (Figure 3); thus, they play similar roles as structural polysaccharides in living organisms: chitin in invertebrates and insects’ cuticles, and scaffolds and cellulose in terrestrial plants [18]. Both biopolymers’ main role is support and protection [19], and the main difference in their molecular structure is the acetamide group on the C-2 position in chitin, instead of the hydroxyl group in cellulose. Chitin is a white, nitrogenous, hard, and inelastic material [13]. It is a linear polymer composed of 2-acetamido-2-deoxy-β-D glucopyranose units. According to the source and the condition of its isolation, chitin is deacetylated to different degrees [20]. In chitin, the degree of deacetylation is usually 0.9, which means it contains 5 to 15% of amino groups that might occur during an extraction procedure [20]. It is similar to the average length of macromolecules in chitin, which is also dependent on its source. 

In 1859, Charles Marie Benjamine Rouger succeeded in turning insoluble chitin into a soluble polymer version by boiling it in a concentrated potassium hydroxide solution under reflux. The new substance, which he named “chitine modifee”, was soluble in dilute organic acids. He was the first to describe the deacetylation of chitin; however, the new substance name “chitosan” was first introduced several decades later by Hoppe-Seyler [2,21]. 

The first industrial production of chitosan started in Japan in 1971 [22]; since then, chitin, and especially chitosan, has found numerous industrial applications in various fields, such as pharmacy, medicine, the food industry, agriculture, biotechnology, waste management, etc. [23,24].

Chitosan is usually obtained from chitin via alkaline hydrolysis of acetamido groups. When the deacetylation degree (DDA) reaches about 50%, chitosan becomes soluble in aqueous acidic media [21,25]. The methods of deacetylation of chitin were already presented in detail in 1973 by Muzzarelli [26]. Several methods have been proposed for the deacetylation of chitin, mainly involving alkali treatment with various combinations of strong sodium and potassium hydroxide solutions (from 30 to 60%), temperatures (from 70 to 140 °C), and treatment times (up to 10 h) [27]. Alternative approaches, which have been proposed in order to increase the DDA and molecular weight of chitosan, and, at the same time, to lower the process alkali concentration and energy consumption [28], involved enzymatic treatments [29], steam explosion [30], low-frequency ultrasound [31], microwave assistance [32], multi stage procedures [33], etc.

The DDA is of crucial importance for chitosans’ activities and applications, such as their antibacterial properties, enhancement of immune activities, lipid lowering, etc. [34]. In general, according to DDA, we are talking about low (DDA) chitosan when its DDA is between 55 and 70%, medium chitosan between 70 and 85%, high chitosan between 85 and 95%, and ultra-high chitosan when its DDA reaches 95 to 100%. In order to achieve chitosan with ultra-high DDA, the application of some special solvents, such as n-butyl-alcohol-sodium hydroxide, amyl alcohol, or dimethyl sulfoxide, were proposed; however, such procedures increase costs and the environmental burden [34]. 

The main functional groups of chitosan are hydroxyl, amino, and N-acetyl groups, which are involved in intermolecular and intramolecular bonding in chitosan’s supramolecular structure [35,36], as well as for any interactions with other substances, materials, and tissues. 

There are two possibilities when forming inter- or intra- molecular hydrogen bonds: between the C3-OH of one and a glycoside group of another chain, or between C3-OH and the oxygen atom of the adjacent chitosan glycosidic ring [35]. 

Chitosan’s molecular activity, solubility, and conformation in aqueous media are strongly influenced by the share of deacetylated glucosamine units and their distribution in the macromolecules [37]. It is the only semi-natural cationic polymer, and, as such, is of extreme interest in some applications, such as: adsorption, flocculation, depollution, bioactivity, etc. The solubilisation of chitosan occurs in acidic media as a result of -NH_2_ functional group protonation and the conversion of chitosan into polyelectrolyte [25]. The best solvent for chitosan is formic acid; however, the most commonly used is acidic acid, at about 1% and pH 4 [38]. The percentage, as well as the distribution of N-acetyl groups in macromolecules, significantly influences chitosan’s p*K*a, solubility, biodegradability, etc. [39,40]. Chitosan solution properties and its molecular conformation depend on the degree of deacetylation, distribution of N-acetyl groups in the chains, and average molecular weight [41], as well as on the acid solution concentration [42]. In dilute chitosan, acidic solutions confirmed semi-flexible rod type conformation of the macromolecules, owing to highly charged polycationic chains in the acidic media [43]. Pedroni et al. reported that, in 0.5 wt% Chitosan in 2% uranyl acetate solution, fully deacetylated macromolecules form a stretched conformation caused by the electrostatic repulsion of -NH_3_^+^ groups, which is intercalated by micelle-like agglomerates formed by the almost fully acetylated molecules [44]. The agglomerates’ radius is proportional to the degree of deacetylation, as the present -NH_3_^+^ groups cause their electrostatic swelling. It was shown by Franca et al. [45] that -NH_3_^+^ groups in neighbouring C2 atoms increase water molecules’ exchange near the O3 atom, and thus destabilise the HO3---O5 hydrogen bond. This is mainly responsible for the predominantly two-fold helical chain conformation. These authors proved, based on solvation energies calculations, that the solubility of chitosan is inversely related to its ability to form intramolecular HO3---O5 hydrogen bonds. As such, chitosan is an important biocompatible and biodegradable polymer with ion exchange, antimicrobial, and haemostatic properties. Chitosan’s molecular structure allows for a variety of derivatisations that have resulted in a wide range of fungistatic, bacteriostatic, drug delivery, fertilising, thickening, stabilising, flocculating, etc., applications. These agents are used in the fields of biomedicine, pharmacy, food industry, agriculture, ecology, etc. [25].

Chitosan’ solubility and molecular conformation in solutions are of extreme importance, as they largely influence interactions, adsorption/desorption kinetics, and, thus, preparation of its solid forms, such as: gels, fibres, films, nano capsules and nanoparticles, functional coatings, etc. 

**Figure 5:**
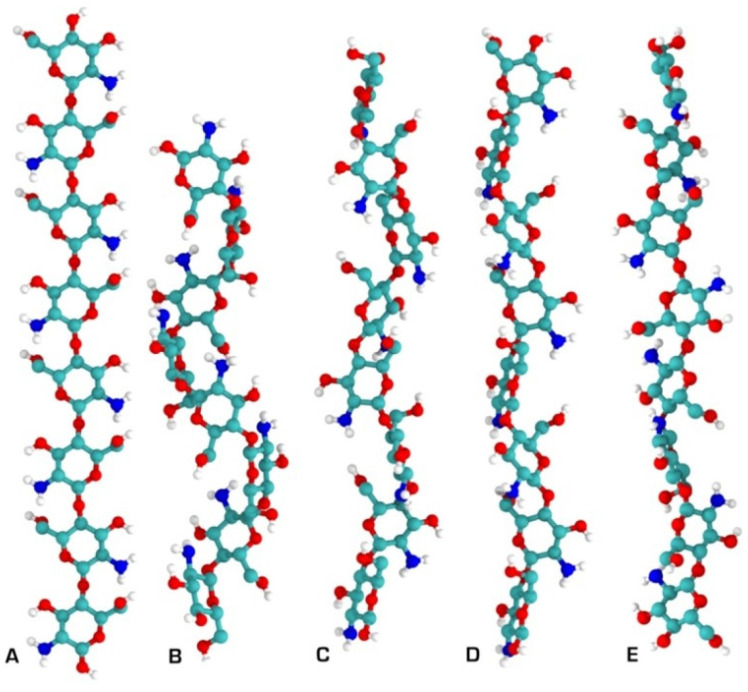
Chitosan secondary structures as determined by solid X-ray crystallography. (**A**) Two-fold; (**B**) three-fold; (**C**) four-fold; (**D**) five-fold; (**E**) two-relaxed-fold [40].

**Figure 6:**
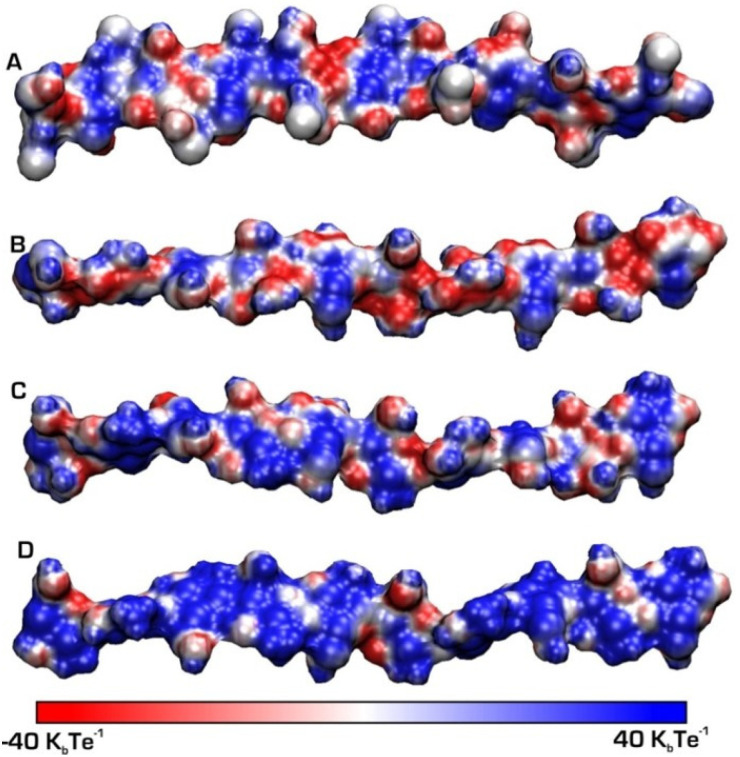
Chitin and chitosan chains simulated at different pH values: (**A**) chitin and (**B**) chitosan at basic (pH = 10), (**C**) chitosan circumneutral (pH = 6.5), (**D**) chitosan acid (pH = 3.5). A positive charge is represented by patches in blue [40].

In the solid state, chitosan’s structure was recognised as similar to that of α-chitin: fibrillar, with a high degree of crystallinity [46]. The first X-ray results on chitosan’s supramolecular structure were published by Clark and Smith [47]. Their structure is widely accepted, and was called tendons chitosan by Ogawa et al. [48]. The X-ray results showed an extended two-fold helix and a zig-zag structure (Figure 5) [40]. Cunha et al. also showed that tuning of chitosan’s electrostatic contributions, and thus its physical state, can be altered easily by a change in pH, which they confirmed through simulation results (Figure 6). The simulations proved the electrostatic similarities between chitin and chitosan in basic media. Ogawa and co-workers also analysed chitosan’s supramolecular structure after heating and removing crystallisation water from the structure. They called the new structure anhydrous allomorph “annealed” chitosan. Chitosan chains crystallise in an orthorhombic crystal unit cell with the dimensions: a = 0.828 nm, b = 0.862 nm, and c (fibre axis) = 1.043 nm, comprising four glucosamine residues and the resulting density 1.52 g/cm^3^ [49]. The chain’s conformation is a two-fold helix (Figure 5) stabilised by intramolecular HO3---O5 hydrogen bonds.

However, in its usual form, obtained by deacetylation of chitin from crabs, chitosan is hydrated, which means that a unit cell beside four chitosan molecules contains eight water molecules [50], which results in changes in the unit cell’s dimensions. There are direct hydrogen bonds between adjacent molecules (N2----O5), which form sheet structures parallel to the *bc* plane. In this structure, water molecules form columns between the sheets and contribute to stabilising the structure by making water bridges between the polymer chains. 

Unfortunately, different forms of solid materials, such as films, fibres, membranes, scaffolds, etc., which are usually produced from partly deacetylated hydrated chitosan, have low mechanical properties, which represents an important obstacle for many chitosan applications. Investigators report on various approaches, such as dissolving, crosslinking, application of plasticisers, annealing, etc., to improve the mechanical properties of solid chitosan materials [51,52,53]. 

Among the various approaches that have been proposed to solve this problem, different combinations of chitosan and cellulose are of particular importance.

## 3. Cellulose 

**Figure 7:**
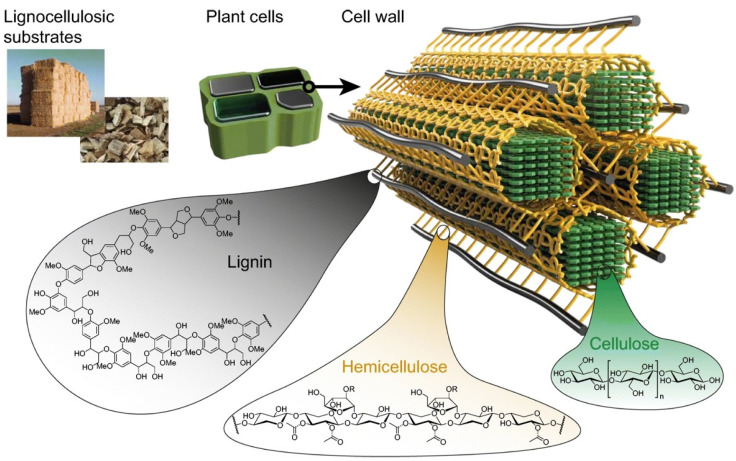
Schematic model of cellulose, hemicellulose, and lignin arrangement in lignocellulosic sources, [56].

As the most abundant natural polymer, cellulose is produced at an annual rate of 10^11^–10^12^ t [54]. It is produced in large amounts by plants, among which cotton fibres are the purest source of plant cellulose. The most abundant source is soft and hard woods, and there are also sources such as annual plants, weeds and bamboo, as well as animal sources such as the sea organism, *Tunicat*, which produces animal cellulose tunicin; microorganisms, which produce bacterial cellulose, such as *Gluconacetobacter Xylinum* strains, etc. [55]. In nature, cellulose typically forms a sort of composite with hemicelluloses, and in woody plants, with lignin (Figure 7). As a natural material, cellulose has served humankind in everyday life for thousands of years in the form of wood, paper, and textile fibres such as cotton, flax, or ramie, etc. However, its applications experienced a real boost in the 1870s, with the discovery of its derivative, cellulose nitrate, and production of the first thermoplastic polymer material celluloid [1]. In this period, the development led to a wide palette of products and applications. After the extraction from its sources and subsequent dissolving, cellulose can be applied as a complex biodegradable, biocompatible polymer with different reactive functionalities for a chemical conversion and new products development, such as fibres, films, nanoparticles, etc. 

**Figure 8:**
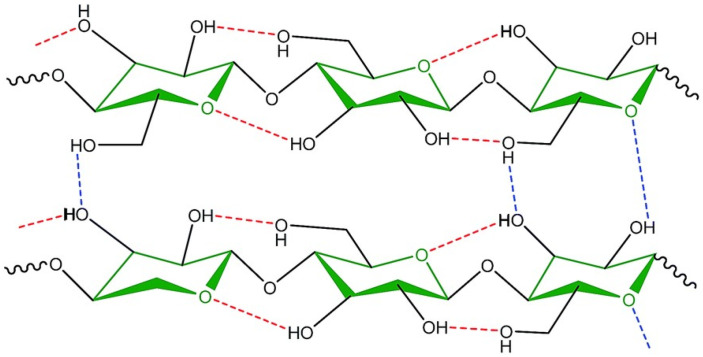
Inter- and intra- molecular hydrogen bonds in the molecular structure of cellulose [58].

Intermolecular and intramolecular bonds in cellulose are of crucial importance for its properties (Figure 8), as it is responsible for the extremely complex and highly crystalline structures built by nature to provide support and protection [57]. 

There are at least four different polymorph supramolecular cellulose structures. The native cellulose I type is the most crystalline, and is composed of Iα and Iβ crystalline phases [54,59]. Cellulose type II is formed by the so-called mercerisation treatment with sodium hydroxide, or during the regeneration process in the production of viscose fibres. Diamine treatment of the cellulose I or II gives the type III_1_ or III_2_, respectively, and the type IV is produced by heat treatment of the type III in glycerol. The elastic moduli of different types of cellulose structure are different, and range from 138 GPa for type I to 75 GPa for type IV, which proves that there are significant differences in the molecular conformation and arrangements of intra- and inter -molecular H-bonds [57,60]. The fact that the elastic modulus of the most famous fibres used in composites, glass fibres, is 78.5 GPa, clears up the importance of natural cellulose fibres as a reinforcement phase in various composites [57,61]. Besides mechanical properties, the cellulose molecular structure influences its main interaction properties, such as hydrophilicity, chirality, biodegradability, and chemical variability. 

Cellulose is the most important structural component of the plant cell wall responsible for its mechanical strength [62], and it has a complex supramolecular structure that has been explored by many scientists; therefore, many different structural models have been proposed [54,63,64]. The models included folded or straight molecules and amorphous crystalline fibrils, or crystalline fibrils with an amorphous surface layer. However, both models showed weaknesses when compared to the properties of real systems. Therefore, the so-called “mesomorphous-crystalline paracrystalline” model of elementary fibrils was proposed, which allows the description of both native and modified celluloses, and predicts their mechanical, thermal, sorption behaviour, solubility, and other properties [65]. This model also explains the possibility of chemical or mechanical production of various forms of nanocellulose, such as nanocrystalline cellulose, nano whiskers, or nanofibrils. All of these forms are elongated, needle shaped, elliptical, or rod shaped [66] and have been shown to be nearly perfect reinforcing components in biocomposites, especially when combined with chitosan [67].

An excellent model system for representation of natural cellulose structure in plants is that of cotton fibres, which are also a rich source of a-cellulose, as, in mature cotton fibres, its content reaches from 90 to 95%. Cellulose microfibrils in mature cotton have a relatively high (63–68%) content of crystalline regions and are composed of 2–3 elementary fibrils with a relatively large cross-section (3.6–4.7 nm). Martinez-Sanz et al. [68] proved that cellulose microfibrils in mature cotton fibres have a core–shell structure with a densely packed inaccessible Iβ crystalline core, with a diameter about 2 nm surrounded by a hydrated paracrystalline shell, which enables moisture to penetrate into the interfibrillar spaces of the relatively densely packed microfibrils.

The discovery and development of suitable solvents and dissolution processes led to a variety of regenerated cellulose solid structures, such as films (cellophane) and fibres such as viscose, cupro, modal, lyocel, etc. These fibres are solid cellulose in pure form, and, as such, they differ from natural fibres in many ways. Most importantly, they have lower wet mechanical properties and higher hydrophilicity compared to natural fibres, owing to the lower DP of cellulose, and hence, lower crystallinity.

Cellulose, with its exceptional properties, could be an excellent substrate for the development of functional biocomposites, in which it can provide hydrophilicity and mechanical properties. As such, it is well established in fields such as packaging, medicine, and hygiene. However, in these applications, antimicrobial properties are of extreme importance, and it has been shown that chitosan can offer a variety of effective solutions here. Combining chitosan with cellulose can provide cellulose with several new functions, such as antimicrobial, antioxidant and antiwrinkle properties [69,70,71]. To improve specific functionalities and neglect the disadvantages of cellulose and primary chitosan, their derivatives have also been prepared and combined to form composites. Therefore, this review includes these derivatives as well. Although numerous chitosan–cellulose composites (and their derivatives) have been developed, there are still many challenges when it comes to manipulating their structure and chemistry to optimise the synergistic effect in terms of specific properties.

## 4. Cellulose–Chitosan Functional Biocomposites

To date, cellulose–chitosan biocomposites have been developed to combine the structure-forming ability, mechanical properties, hydrophilicity, and water stability of cellulose with the chitosan’s electron donors, and, of course, its antimicrobial properties [67]. They could be prepared through processing of bulk, such as, for example, via homogeneous blending in dissolved or solid form [72], or by adsorption or coating processes, usually using chitosan as the adsorbate, and cellulose, in the form of fibres or films, as the substrate [73,74]. In general, it was found that increasing the cellulose content improved the mechanical strength and increasing the chitosan content improved the antibacterial and biocompatible properties of the composites [67].

Similar chemical structures of cellulose and chitosan predict their compatibility and the possibility of their blending [75]. The intermolecular interactions between cellulose and chitosan were investigated thoroughly by Holmberg and co-workers 25 years ago [6]. They confirmed the intermolecular interactions between the molecules of the two polymers, and found that, between the cellulose surface and the chitosan-coated surface in a dilute electrolyte solution, long-range attractive forces arise, most probably due to bridging if oppositely charged Coulomb interactions are the driving force for their interactions.

Blended cellulose–chitosan composites can be obtained from solutions in two different ways: by dissolving each biopolymer separately in typical solvents and then mixing the two solutions, or by mixing the solid polymers and dissolving both in the same solvent [75]. The second approach is more reasonable, but it is not so easy to find the right common solvent for both biopolymers. Recently studied common solvents for cellulose and chitosan include N-methylmorpholine-N-oxide (NMMO), ionic liquids, and ethylene diamine/potassium thiocyanate, which are usually expensive and difficult to remove from the material. On the other hand, the use of green solvents to dissolve cellulose and chitosan directly is still a problem [76]. 

### 4.1. Structures and Forms

To take full advantage of all the distinguished chitosan and cellulose functions, such as appropriate mechanical properties, large accessible surface area and well-defined porosity, high functionality, etc., investigations focus on the development and application of a variety of structures and forms of cellulose–chitosan composites, mainly aerogels [77,78,79], foams or sponges [80,81], membranes [82], hydrogels and films [83], nanoparticles [84,85], fibres and nanofibers [86,87], etc. Many different approaches and techniques have been applied and studied, including various solubilisation processes, as well as drying techniques for aerogels formation, such as freeze-drying, supercritical conditions, vacuum, ambient pressure, microwaves, etc. [88]. 

The most commonly used or so-called conventional methods for the production of polymer composites are extrusion, injection, and moulding of polymer blends or co-extrusion of two or more different polymers, which include a variety of methods for the production of films, fibres, and membranes [89]. These methods are particularly well suited to thermoplastic polymers, as long as the processing temperatures are high and allow the desired shape to be achieved after cooling. When biodegradable polymers are used, some of these methods are not suitable due to polymer degradation. More common methods in these cases include solvent casting, phase separation such as gelation, freezing or drying, etc. [67]. Cellulose solutions have been spun by the viscose process for centuries to produce regenerated cellulose fibres, and initial attempts to produce cellulose–chitosan composite fibres were also conducted in this way [90]. However, in recent decades, more and more attention has been focused on the use of electrospinning processes to produce nanofiber composite structures from cellulose and chitosan [91]. 

Another effective approach to combine cellulose and chitosan is to form thin layers and/or coatings, usually on cellulose as a solid macroscopic substrate. Coating processes aim to transfer liquid onto a solid material to create a surface film, ensuring its high availability at the surface. The simplest forms of coating rely on the temperature, viscosity, pH, concentration, and ionic strength of the liquid to control the thickness and uniformity, as well as the stability of the coating. With all these physicochemical parameters and possible pretreatments (e.g., with plasma), the coating can be manipulated to achieve a permanent bond, or an uncontrolled or controlled release. Various technological methods can be used for coating, which have different economic and environmental impacts. The most useful for applying chitosan to cellulose matrices are roll-to-roll coating, dip coating (soaking and impregnation), screen and pad printing, spray coating, and electrospraying.

Electrospraying is a technique used to generate micro/nanospheres as coatings, and it was applied successfully for the synthesis of chitosan-based micro and nanospheres’ production [92]. Apart from being a high yield technique, electrospraying has the advantage of not requiring an external dispersion/emulsion phase, which often contains components that are undesirable for biomedical applications. It can also be used to produce quite thin and homogeneous coating films.

#### 4.1.1. Hydrogels, Aerogels, Sponges, and Membranes

All these structures are defined as porous and cellular networks. Among the important applications of these structures is the removal of various types of pollutants, such as dyes, heavy metals, proteins, oil, etc., from water. In the review article of Shen et al. it was presented that much fundamental research has been conducted on the preparation of cellulose and chitin hydrogels, including the development of solvent systems for native cellulose or chitin, hydrogel formation techniques, physical or chemical cross-linkers, and drying methods, combining both polymers, etc. However, studies have focused mainly on potential applications of the hydrogels in water purification and pharmaceutical and medical systems, and less attention has been paid to other areas, such as their use in the electronic and optical fields, which has been demonstrated recently [93]. Weng et al. prepared a composite nanofiltration membrane of cellulose and chitosan via interfacial polymerisation of piperazine and trymesoil chloride [82]. Such improved membrane showed a high rejection rate in aqueous dye-salt solutions, and good performance under various pressure conditions. Chen et al. [94] showed that, currently, nanocellulose and composite aerogels provide a highly attention-catching platform for a wide range of functional applications in various fields, e.g., adsorption, separation, energy storage, thermal insulation, electromagnetic interference shielding, and biomedical applications.

**Figure 9:**
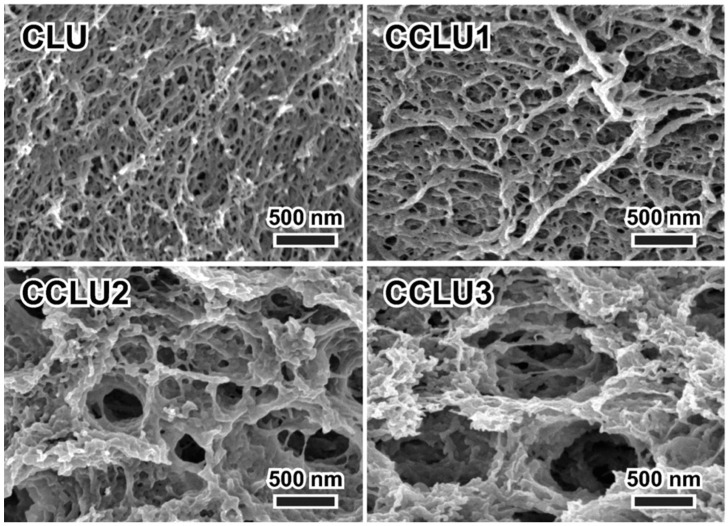
Porous structures of freeze-dried gels prepared using an aqueous LiOH/urea solution for co-dissolution of cellulose and chitosan (CLU: pure cellulose, CCLU1-3 hydrogels with increased amounts of chitosan from 25 to 75%) [96].

Often, ionic liquids are applied when talking about co-dissolution of both polymers. A biocomposite hydrogel of cellulose and chitosan was prepared by dissolving both biopolymers in a 60 wt% aqueous solution of LiBr. The authors reported that chitosan not only provided a functional surface for the adsorption of metal ions from water, but also improved the mechanical properties of the hydrogel [95]. In another study, cellulose–chitosan gels were prepared using an aqueous LiOH/urea solution for co-dissolution [96]. After the cellulose and chitosan were dissolved via a freeze–thaw process, the hydrogels were prepared by regeneration in methanol and washing with water. Increasing the chitosan content changed the porous structures of the gels significantly, as their surface areas increased gradually from 217 m^2^/g for pure cellulose to 337 m^2^/g for the gel containing 75% of chitosan (Figure 9). It also reduced the mechanical strength, while the adsorption capacity of the anionic dye was improved greatly. Furthermore, Rizzo et al. developed the bio-based ionic liquid cellulose–chitosan gels as polymeric soft materials for the desulphurisation of fuel [97].

Saad et al. prepared environmentally friendly imprinting and non-imprinting composites from chitosan and *Ulva lactuca algae* (Alg) and used them to remove Cd(II) ions from aquatic media [98]. They found that the maximum removal capacity for the studied biocomposites was achieved at an optimum pH of 5.5, a contact time of 120 min, a temperature of 25 °C, and a biocomposite dose of 0.1 g.

The wide application fields of porous structures, such as hydrogels, sponges, and membranes, include wound management, tissue engineering, and controlled drug delivery. The work of Wang et al. [99] presented a simple method for preparing redox-responsive hydrogel composites by incorporating chitosan microspheres into cellulose derivate as carboxymethylcellulose using the di-sulphide crosslinker cystamine dihydrochloride. In vitro, accelerated drug release was shown in weakly acidic or reducing media. The in vitro cytotoxicity and cell apoptosis studies showed that the drug-loaded composites enhanced the inhibition of HepG2 cells in the presence of glutathione. In addition, the composites showed excellent antimicrobial activity against *E. coli* and *Staphylococcus aureus.* Qinghua Xu et al. reported the preparation of a novel nanocomposite hydrogel based on cellulose nanocrystals and chitosan to be used as a carrier for the controlled delivery of theophylline [100]. The cumulative percentage of drug release of the composite hydrogel was approximately 85% and 23% in gastric (pH 1.5) and intestinal (pH 7.4) fluids, respectively. In their study, Huang et al. pointed out a dissolvable self-healing hydrogel based on chitosan derivate as carboxymethylchitosan and cellulose nanocrystals for healing deep partial thickness burn wounds [101]. Cytotoxicity tests and three-dimensional cell cultures demonstrated the excellent biocompatibility of the hydrogel and its ability to support cell growth as an extracellular matrix. A unique biodegradable, super porous, swellable, and pH-sensitive nanocellulose reinforced chitosan hydrogel with dynamic mechanical properties was prepared for oral administration of curcumin suggested to improve the bioavailability of curcumin for absorption from the stomach and upper intestinal tract [102]. Further, hydrogels based on chitosan and oxidised carboxymethylcellulose, optimised for controlled release of curcumin and used in the treatment of skin diseases, were developed by Dellali et al. [103], and showed a positive antioxidant, antimicrobial, and proliferation impact. Hasan et al. [104] prepared a bacterial cellulose–chitosan crosslinked hydrogel and loaded it with nitric oxide in the form of polyethyleneimine diazeniumdiolate (PEI/NO) for the treatment of polymicrobial wound infections. In this way, the improved NO loading enhanced bactericidal efficiency, and the sustained drug release lasted four days. 

**Figure 10:**
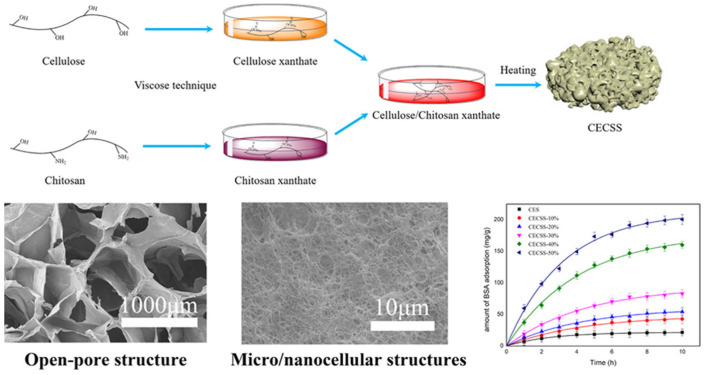
Schematic presentation of cellulose chitosan composite sponge formation with the micrographs of the sponges’ open-pore structure and the influence of chitosan content onto BSA adsorption efficiency. Reprinted (adapted) with permission from Liu, C.; Yu, J.; You, J.; Wang, Z.; Zhang, M.; Shi, L.; Zhuang, X. Cellulose/Chitosan Composite Sponge for Efficient Protein Adsorption. *Industrial & Engineering Chemistry Research* 2021, 60, 9159–9166 [81]. Copyright 2021 American Chemical Society.

Liu and co-workers, on the other hand, developed sponge and foam structures for protein adsorption using the viscose process [81]. In this case, the unique porous structure (Figure 10) and reactive groups of chitosan enabled high adsorption capacity for bovine serum albumin (BSA).

Another huge application field for antimicrobial and antioxidative biopolymer materials in the form of hydrogels is food packaging and food shelf-life improvement. In particular, materials such as mesoporous hydrogels have been studied extensively [105,106,107]. In the chapter of the book *Cellulose-based Hydrogel Films for Food Packaging* [108], the potency of cellulose-based hydrogels as substitutes for food packaging materials was highlighted. In addition, it is noted that the combination of other polymer materials with cellulose gives the hydrogel tailored properties to serve as a food packaging material. The cellulose-based hydrogel is able to store fresh fruits and vegetables that trap or release moisture because the hydrogel has the ability to trap moisture. The potential of this strategy lies in the combination with chitosan in the right ratio, concentration, and pH.

Miyajima et al. [109] prepared porous carbons through the K_2_CO_3_ activation of chitosan and cellulose, and estimated their potential as a freshness-preserving adsorbent for fruits and vegetables. The pore structures, the water vapour, and the ethene adsorption behaviours were investigated thoroughly, and it was found that the N and O-containing functional groups in chitosan-based carbons provided high amounts of water adsorption sites, while the carbon’s ultramicropores were suitable for the ethene molecule adsorption, which was comparable to that of a commercial ethene adsorbent.

#### 4.1.2. Films and Coatings

The most important areas of application for biopolymer films and coatings are the packaging industry, the food industry (edible films and coatings), and medicine (wound dressings, drug delivery) [110]. In addition to specific surface functionalities, the optimal mechanical properties of materials are also of the utmost importance, especially in packaging. In order to achieve the latter, it is crucial, especially in the case of cellulose–chitosan blended films, to achieve homogeneous structures without obvious phase separation. Lin et al. achieved this using ZnCl_2_.3H_2_O as the solvent [111]. This research also showed that the solvent had no effect on the antimicrobial properties of the blended films. Zhou et al. [112] succeeded in preparing antibacterial and homogeneous cellulose–chitosan composite films by dissolving both biopolymers in the ionic liquid 1-ethyl-3-methylimidazolium acetate. A homogeneous and compact structure of the films and a more thermally stable structure compared to regenerated cellulose or chitosan films were obtained, as well as an antibacterial effect on *E. coli* and *S. aureus*. 

Blending of different proportions of separately dissolved cellulose and chitosan in LiOH/urea through freeze–thawing and regeneration in water/ethanol was one of the recent approaches used to develop environmentally friendly processes for nanocomposite cellulose–chitosan films [113]. Different contents of chitosan allowed tuning of the pH response of the films, as well as the slow release of chitosan. Examination of the composition of the films confirmed finely built microstructures based on hydrophobic interactions. It was also found that the addition of chitosan reduced the mechanical properties of the films by disturbing the lamellar structure. 

Because of these problems, many approaches still focus on using cellulose as a physiochemically solid substrate and forming chitosan layers on top. The main advantage of these approaches is, on the one hand, the better availability, control, and preservation of the functionalities of the chitosan, and, on the other hand, the preservation of all the previously mentioned excellent properties of the cellulose substrates, such as fibres, films, paper, membranes, etc.

To understand the cellulose–chitosan interactions better, the group of Janne Laine thoroughly studied the pH-dependent adsorption behaviour of chitosan on a cellulose model surface using a quartz crystal microbalance with dissipation (QCM-D) [7]. The molecular-level interactions between the adsorbed chitosan layers were studied using atomic force microscopy (AFM) and colloidal probe force measurements in the liquid phase. The adsorption of chitosan increased with a pH below the solubility limit of the polymer. The adsorption behaviour could not be explained based on electrostatic interactions alone, as there was a specific interaction between the polymers. The swelling and viscoelastic properties of the adsorbed chitosan layer were influenced strongly by the pH. At high pH, the layer swelled and became more elastic due to the insolubility of the chitosan. Colloidal probe force measurements showed an increase in electrosteric repulsion after adsorption of chitosan at pH 5. Above the solubility limit of chitosan, at pH 7, the pull-off force and its range increased significantly compared to lower pH values, indicating that wet adhesion increased between the chitosan-coated cellulose surfaces. The results presented are very important when it comes to understanding real systems, such as cellulose fibres and films coated by chitosan.

In an earlier work from 2010, Da Roz et al. reported the adsorption of an ultrathin layer of chitosan on spin-coated cellulose films, where the efficient attachment was induced by the oxidation of cellulose, which provided anionic sites for electrostatic interaction with the positively charged chitosan [73]. The adsorption of a chitosan layer on the cellulose film also resulted in chemical and morphological changes with the introduced amino groups and increased the film’s roughness. The successful adsorption of chitosan on cellulose opens the way for several applications where the obtained properties of biocompatibility can be combined with antimicrobial or bactericidal activities. 

The main research question in these studies is the balance between durability and functional efficacy. In terms of the main antimicrobial functional units of chitosan, the amino groups, are, in many cases, also cellulose–chitosan binding sites, and improving the durability of the layer usually decreases the antimicrobial efficacy due to the lower availability of amino groups [114,115].

The investigations into the chitosan adsorption onto different kinds of cellulose fibres, natural or regenerated, revealed many advantages that the combination of cellulose and chitosan could provide. The main objective here is to create the most resistant and durable chitosan layers possible, while maximising the antimicrobial efficacy of the fibre surfaces without altering or even improving other properties. A variety of application techniques were used, such as adsorption of chitosan from a solution onto cellulose fibres, [116], application of chitosan nanoparticles distributed in a chitosan macromolecular solution [85], the layer-by-layer technique [117], cross-linking, UV irradiation [118], etc.

Cheng et al. synthesised the chitosan derivative 1-hydroxymethyl-5,5-dimethylhydantoin-chitosan, which was applied to cotton fabrics with 1,2,3,4-butanetetracarboxylic acid (BTCA) as a crosslinking agent [119]. The treated cottons exhibited excellent antimicrobial activity, the wrinkle recovery of the treated fabrics improved, while the breaking strength decreased compared to that of uncoated cotton. In another study [120], phosphorylated chitosan was used as an intumescent flame retardant, which was fixed on the fibre surface by electrostatic interaction and chemical grafting, preserving the tensile strength of the fibres and giving the cotton permanent flame-retardant properties with potential antimicrobial activity. In addition to antimicrobial activity, these studies also focused on the potent antioxidant activity demonstrated by the ABTS assay. A similar non-toxic procedure was described by Alonso et al. [121] for the grafting of chitosan-based microcapsules containing grapefruit seed oil extract onto cellulose fibres. The fibres were previously UV irradiated and then functionalised from an aqueous emulsion of the chitosan. The essential oil and materials showed 100% inhibition of *E. coli* and *S. epidermidis* up to 48 h of incubation. 

As a fibrous cellulose substrate with a large surface area, viscose fibres or non-wovens are often used in medical and hygienic fields as pure cellulose material with high hydrophilicity, but, at the same time, a lack of antimicrobial properties. Over the past decade, our research group has conducted studies on the specific functionalities that can be introduced into viscose cellulosic fibres through various chitosan treatments. In one such study, gynaecological tampons were prepared via the adsorption of chitosan onto viscose tampon-based fibres. It was found that the tampons prepared in this way exhibited high hydrophilicity, antibacterial and antifungal activity, and maintained a physiological pH [122]. Further studies have been conducted to improve and extend the antimicrobial activity of chitosan, while its disadvantage is that it is more active against Gram-positive pathogenic bacteria and less so against negative Gram-pathogenic bacteria. Moreover, chitosan is a poor antioxidant; thus, antioxidative properties have been achieved by binding or encapsulating some other substances such as flavonoids, curcumin, iodine, and eugenol [123,124,125,126]. Ristić et al. showed that quaternary chitosan is more effective than primary chitosan as a cellulose coating because it contains quaternary amino groups that are not protonated pH-dependent [127].

Another form of chitosan that has been studied in detail with respect to surface coatings are nanoparticles. It has been shown that chitosan nanoparticles can be a successful delivery system, due to their larger specific surface area and controlled delivery of extracts and polyphenols, so our group’s work has been extended to drugs and metal ions. To improve the antimicrobial and antioxidant activity of chitosans, chitosan nanoparticles with embedded iodine and zinc ions and drugs were applied to the different pretreated viscose fibres [128,129]. Ristić et al. applied the encapsulation of a model drug in chitosan and its water-soluble N,N,N-trimethyl derivative. This nanoparticle dispersion was then attached to viscose cellulose fibres to create a potential fibrous drug-delivery system for gynaecological use, i.e., tampons as antimicrobials themselves, or as drug reservoirs. It has been shown that the antimicrobial activity is even better, and a short time-controlled release of the drug has been achieved [128]. Milanović et al. found that viscose fabrics modified by chitosan–iodine or chitosan–zinc nanoparticles’ dispersion was a very promising functionalisation process with antimicrobial and antioxidant properties. However, to provide good coating stability, several pre-activation procedures were needed, such as plasma activation and TEMPO oxidation [130]. 

**Figure 11:**
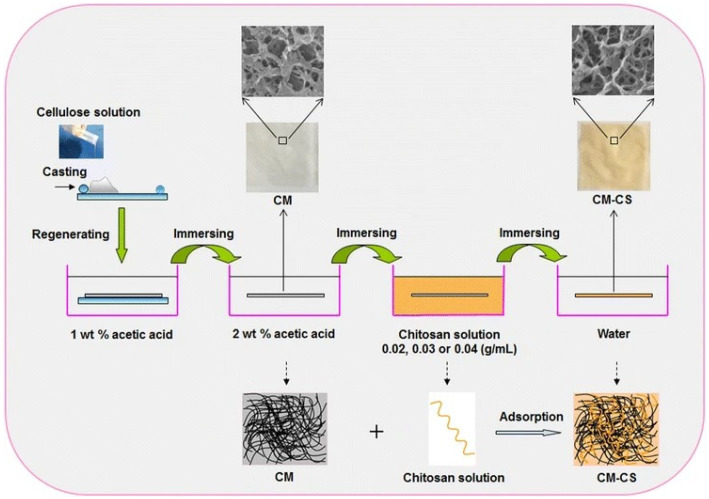
Schematic presentation of the approach of Cao et al. to the preparation of composite chitosan coated cellulose membrane Reprinted by permission from: [Springer Nature] [*CELLULOSE*] [131] (A facile and green strategy for the preparation of porous chitosan-coated cellulose composite membranes for potential applications as wound dressing, Cao, Zhenni, Luo, Xiaogang, Zhang, Hao, Fu, Zhen, Shen, Zhi, Cai, Ning, Xue, Yanan, Yu, Faquan), Copyright (2016).

It turns out that chitosan coatings are usually strongly focused on bioactive applications. In addition to functional fibres, other structures may also be of interest. Cao and co-workers fabricated a cellulose–chitosan wound dressing membrane by adsorbing chitosan onto a cellulose membrane made from cotton linters [131]. The membrane had a 3D network structure with micro- and nano-sized pores, which allowed swelling and a large accessible antimicrobial surface area for the chitosan (Figure 11). 

Cellulose produced by bacteria—bacterial or microbial cellulose—has seen increasing consideration as an excellent material for packaging or medical applications, as well as for clothing and accessories, due to its biocompatibility, edibility, non-toxicity, high tensile strength, high water-absorption capacity, etc. [132,133]. However, bacterial cellulose itself has no antimicrobial activity, and chitosan has been the solution and the missing element in many attempts to produce antimicrobial cellulose–chitosan films [134]. Due to the specific (fibrous) porous structure of bacterial cellulose–chitosan composites, the terms “membranes” or “hydrogels” are also often used for these materials instead of “films”. The investigation conducted by Phisalaphong et al. revealed that the addition of low-molecular-weight chitosan into culture medium of *A. xylinum* during biosynthesis caused a significant increase in the tensile strength, surface area, and water sorption capacity of films, as well as smaller pore diameters and a denser fibril structure. However, with dilute concentrations of added chitosan, which amounted from 0.25 to 0.75% (*w*/*v*), the films did not achieve significant antimicrobial activity [135]. On the other hand, a study by Savitskaya et al. reported the successful integration of chitosan into a bacterial cellulose fibrillar structure, where 0.6% (*w*/*v*) of chitosan caused significant inhibition of *E. coli*, *S. aureus*, and *P. aeruginosa* [136]. Later, some other researchers showed that the antimicrobial activity of bacterial cellulose chitosan composites depend strongly on the molecular weight of the added chitosan [137]. Therefore, there are many other approaches which use combinations of different formulations together with chitosan to improve its antimicrobial effects. Specifically, for wound dressings, many different formulations and approaches have been applied to improve their antibacterial activity, such as: silver formulations [138,139], zinc oxide [140], nitric oxide [104], ferulic acid [141], etc. Through carboxymethylation and selective oxidation of bacterial cellulose and cross-linking with chitosan, Xie et al. produced a cellulose–chitosan wound dressing that inhibited bacterial proliferation and exhibited an 80% wound-healing rate after three weeks. The presence of carboxyl groups and the amorphous arrangement of the chitosan chains on the surface increased the antimicrobial properties of the composites. The authors found that this particular surface structure of the composites can attract bacteria, which they termed a “directed adhesion effect” [142]. Another recent study [143] evaluated the antibacterial impact of bacterial cellulose (BC) including dendrimer, chitosan, henna, green tea, and Malva sylvestris in various bacteria such as *S. aureus* and *P. aeruginosa*, and it was pointed out that the antimicrobial effect of cellulose increased due to using chitosan, dendrimer, and the herbal materials.

#### 4.1.3. Fibres 

The 1990s were the most active period for research on the production of cellulose–chitosan blend composite fibres. Most researchers in the field at that time focused primarily on ways to modify the conventional viscose production process by adding chitosan to achieve antimicrobial activity and maintain all other essential properties of the resulting composite fibres. One of the first such fibres was Chitopoly, which was developed by Seo et al. by mixing chitosan microparticles and viscose solution [90,144]. The composite fibres Crabyon^®^ containing, besides cellulose, about 10% of chitosan, were also put on the market in the late 1990s. The first report about these fibres was recorded in 1999 [145] and many research groups investigated their application properties [146,147,148,149]. Many attempts and modifications of the dissolution and blending processes were reported from that time on. Some of them used chitosan derivatives such as N-acylchitosan in the conventional viscose process [150] or lyocell process with NMMO/H_2_O as a solvent [151]. 

**Figure 12:**
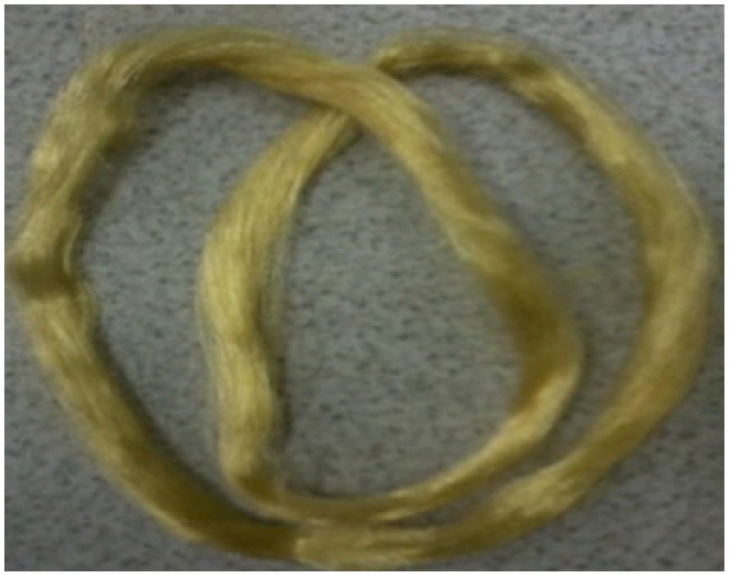
The photograph of the cellulose–chitosan composite fibres made by [152] using a binary ionic liquid system and the dry-wet spinning procedure. Reprinted from *Carbohydrate Polymers*, 88/1, Ma, Bomou, Zhang, Meng, He, Chunju, Sun, Junfen, New binary ionic liquid system for the preparation of chitosan/cellulose composite fibers, 347–351, Copyright (2012), with permission from Elsevier.

As mentioned before, in the last two decades, ionic liquids have been recognised as green and appropriate for cellulose chitosan blend solutions’ preparation, and, as such, they are also very welcome to replace volatile substances in conventional cellulose fibres’ production processes. Ma and co-workers used a binary system of ionic liquids for the dissolution of cellulose and chitosan in one step, as well as to produce composite fibres through the dry–wet spinning process [152]. The resulting fibres (Figure 12) contained 9.4% of chitosan, and it was distributed on the surface as well as in the bulk. The authors pointed out that sufficient stretching of fibres is necessary for excellent mechanical properties of the fibres.

Recently, researchers have focused on specific functionalities and/or applications of cellulose–chitosan composite fibres. Zhuang and his group combined cellulose and chitosan in a wet spinning process and manufactured a fibrous composite with a large surface area and about three times higher adsorption capacity for Co(II) in comparison to pure chitosan [153]. They used separate dissolution processes for both polymers, for cellulose LiOH/urea/H_2_O, and for chitosan LiOH/KOH/urea/H_2_O and mixed both solutions thereafter. They used lab wet spinning equipment for fibre formation.

**Figure 13:**
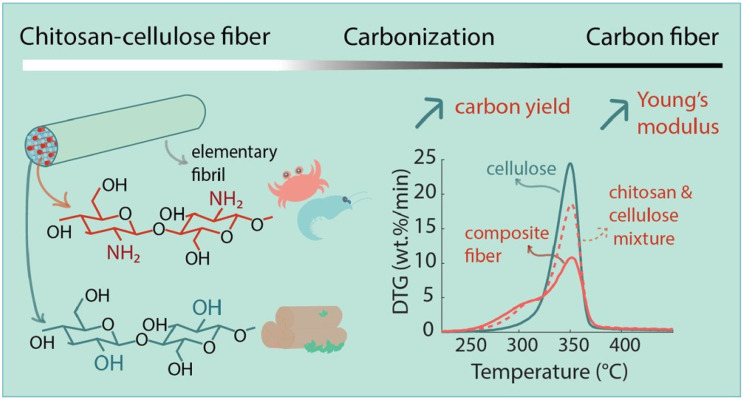
Preparation scheme of regenerated cellulose–chitosan fibres for improved carbon yield and structural properties of the respective carbon fibres [154].

An interesting achievement was also reported by Zahra et al. [154], who prepared blended cellulose chitosan fibres via a conventional dry-jet wet spinning process in order to prepare a precursor for carbon fibres’ production with improved carbon yield. Ionic liquid was used for the direct dissolution of chitosan and cellulose. First, a suspension of chitosan powder in ionic liquid was prepared, and then dissolved by stirring it for 1 h at 75 °C, and then cellulose pulp was added and the blended solution mixed for 2 h. Chitosan presented 25% (*w*/*w*) of the total polymer concentration (Figure 13). The authors compared the properties of the blended fibres to pure regenerated cellulose fibres and found that the homogeneous distribution and tight packaging of the chitosan and cellulose enabled synergistic interaction during the pyrolysis, which led to increased carbon yield and preserved the mechanical properties of the resulting fibres.

**Figure 14:**
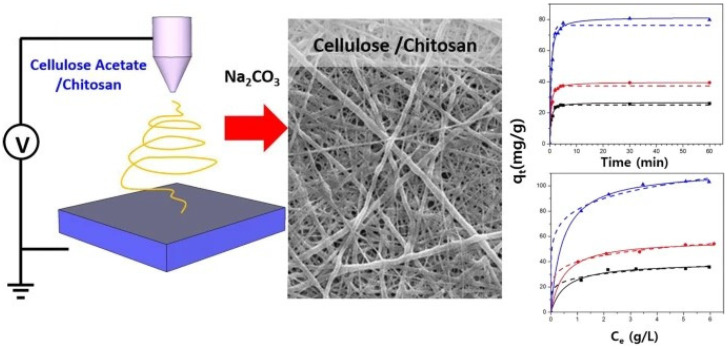
Cellulose chitosan composite nanofibres made via electrospinning from a trifluoroacetic and acetic acid co-solvent system and treated further with Na_2_CO_3_. Reprinted with permission from: Springer Nature, *CELLULOSE* [156] (Fabrication of electrospun chitosan/cellulose nanofibers having adsorption property with enhanced mechanical property, Phan, Duy-Nam, Lee, Hoik, Huang, Bijun, Mukai, Yasuhito, Kim, Ick-Soo) Copyright (2018).

In recent decades, electrospinning has become very popular for producing nanofibres, especially when a large active surface area of the material is desired. This property makes them very attractive for applications such as filters, sensors and affinity membranes, medical textiles, etc. Electrospinning is also an important method of bulk processing, by which cellulose–chitosan composite nanofibrous structures can be provided [155,156]. Manufacturing of composites via electrospinning can be achieved by simply mixing polymer solutions before spinning of the nanofibres, by surface functionalisation after spinning, or by using core–shell electrospinning systems [157,158]. One of the early attempts to electrospin non-derivatised chitosan cellulose fibres was that of Park et al. [159]. As a common solvent for both biopolymers, they used ionic liquid (1-ethyl-3-imidazolium acetate). The composite fibres were electrospun directly into an ethanol co-solvent to remove all ionic liquid from the fibres. In this way, the authors showed that due to their great antimicrobial activity, the produced composite nanofibres are suitable for the treatment of wounds. Many other examples may be found where the chitosan or cellulose derivatives were used to produce composite nanofibrous forms. Thus, in 2008, a simple approach was developed by Du and Hsieh to produce cellulose/chitin/chitosan nanofibres with a variety of compositions, by electrospinning their ester derivatives, cellulose acetate and dibutyrylchitin (DBC), followed by alkaline hydrolysis to cellulose and chitosan [155]. Electrospinning of mixtures of ester derivatives of cellulose and chitin, followed by aqueous hydrolysis, has been shown to be a facile approach to produce cellulose–chitin or cellulose–chitosan hybrid nanofibers. The direct electrospinning of a non-derivatised cellulose chitosan composite nanofibre was, on the other hand, investigated by Devaryan and co-workers [160]. Cellulose from two different sources, i.e., cotton or bamboo, was electrospun with chitosan using an atrifluoroacetic acid and acetic acid mixed solvent. With the further treatment of the fibrous composite with an alkaline–alcohol solution, they produced a water-resistant non-woven form with good mechanical stability. The same co-solvent system of trifluoroacetic and acetic acid was applied for cellulose acetate and chitosan dissolution by Phan and co-workers [156]. They conducted further treatment of fibres using Na_2_CO_3,_ and the resulting nanofibres exhibited better mechanical properties and adsorption capacity in comparison to pure chitosan or pure cellulose nanofibre mats (Figure 14). Moreover, the presence of chitosan in the composite nanofibres enabled metal ions’ adsorption, and, therefore, the authors proposed wastewater treatment applications. 

Another attempt to develop a chitosan–cellulose acetate nanofibrous composite membrane for Cd^2+^ adsorption and wastewater treatment was made by Aquino et al. The investigation revealed that the introduction of chitosan into the composite increased the adsorption of Cd^2+^ ions significantly, by about 65% [161]. 

A useful strategy for designing cellulose chitosan composites using electrospinning is the deposition of the electrospun (chitosan) nanofibresonto different (cellulose) substrates, such as films, membranes, or fibrous forms such as fabrics and non-wovens, etc. Nawalakhe and co-workers [162] developed such a composite for wound dressings by electrospinning chitosan nanofibres on cotton gauze substrates pretreated with atmospheric plasma. Chitosan nanofibres were spun from trifluoroacetic acid, and caused significantly higher surface-antimicrobial activity in comparison to pure cotton gauze. Chitosan nanofibres also improved the absorbency of cotton gauzes by more than 50%, which likely improved the absorption of blood and wound exudates. In a study by our group [163], chitosan particles with embedded catechin and with entrapped resveratrol were mixed with polyethylene oxide and used in an electrospinning device to produce nanofibres/nanocoatings that were applied to the viscose matrix material. The fabricated nanofibre composites deposited on viscose material exhibited excellent antimicrobial and antioxidant activity. Moreover, controlled release of the active ingredients was enabled. The developed nanofibre composites, consisting of chitosan and entrapped (poly)phenolic substances such as catechin or resveratrol, together with viscose nonwovens, have shown high potential for the development of medical textiles for the treatment of damaged or diseased skin. 

Zhiming and co-workers [164] performed an interesting experiment, in which they used sulphated bacterial cellulose sulphate as a substrate for electrospinning of chitosan nanoparticles with an ethyl cellulose ethanol solution. In this way, a heparin-like chitosan ethyl cellulose composite membrane layered onto a bacterial cellulose sulphate membrane was prepared. In this spinning system, positively charged chitosan in the spinning solution and negatively charged bacterial cellulose sulphate membrane on the collector increased the electrostatic force, and the electrospinning of ethyl cellulose became easier. It was also found that during the electrospinning process, chitosan nanoparticles were stretched to nanofibrous forms. The composite membrane exhibited good blood compatibility and improved inflammatory response. Table 1 summarises the main research and development work on the structures and manufacturing processes of cellulose–chitosan biocomposites and their potential applications.

## 5. Conclusions and Future Perspectives 

Cellulose and chitosan have emerged as renewable materials with numerous potential applications. The new era of replacing non-renewable materials with renewable ones is making its way into all aspects of human life. Moreover, the application of multifunctional materials has enabled rapid scientific innovation in most fields, especially when they come into contact with other materials and/or tissues (the human body or food through packaging) and must respond to unpredictable environments. For this reason, composites of cellulose and chitosan (and their derivatives) are of great importance. These composites can be designed at the molecular level and then assembled at the nano, micro and macro levels, depending on what they are to be used for. In this sense, aerogels, hydrogels, sponges, membranes, fibres, and films can be developed. On the other hand, various structures (usually cellulose) can be coated with (usually chitosan) thin films, nanofibres, or nanoparticles. This wide range of structures provides different properties for a variety of high-tech applications, such as packaging, medical devices, advanced sorbents and philtres, etc.

Although the number of research studies and publications in the field of developing new materials by combining cellulose and chitosan has increased greatly since 2011, many questions still remain. As mentioned above, the development of biocomposite materials based on cellulose and chitosan mainly occurs two directions. One is the preparation of intimate blends of the two biopolymers, and the other is the preparation of chitosan coatings, thin films, nanoparticles, or nanofibres on cellulose substrates such as films, membranes, textiles, paper, etc. Both approaches have advantages and disadvantages. The main problems related to the first approach are the selection of a suitable, environmentally friendly solvent and the control of the extent of interactions between the two polymers, which have a crucial impact on the functionality (bioactivity) of the produced composite. In the second approach, where chitosan is usually deposited in the form of nanoparticles, nanofibers, or thin films on a cellulose substrate, more effective functionality is usually achieved as the surface area is increased. However, the main problem in many applications is the poor mechanical or chemical resistance of these coatings.

Therefore, to achieve further improvements, additional derivatizations of the two polymers will most likely need to be investigated and combined more thoroughly. The development of new composites requires a deeper understanding of the interactions between the two polymers in order to preserve all the great properties of both polymers and achieve new advanced functionalities with their synergy. Finally, the materials and processes still need to be adapted for industrial, large-scale production.

## Figures and Tables

**Table 1:** An overview of the research and development of structures and preparation processes of cellulose–chitosan biocomposites and their potential applications.

Structure/ Form	Process	Application	Reference
Aerogels	Blending	Adsorption, separation, cleaning, energy storage	Li et al., 2020 [77], Liu et al., 2021 [78], Yan et al., 2021 [79], El-Naggar et al., 2020 [88], Chen et al., 2021 [94]
Sponges/Foams	Blending/co-dissolution viscose process	Adsorption/medical applications	Kim et al., 2019 [80], Liu et al., 2021 [81]
Membranes	Interfacial polymerisation Bacterial cellulose co-biosynthesis with chitosan	Cleaning, filtration medical/wound management	Weng et al., 2020 [82] Phisalaphong et al., 2008 [135]
Hydrogels Porous carbon structures	Blending/co-dissolution ionic liquids/cross-linking	Desorption/removal of heavy metals desulphurisation of fuel removal of Cd(II) wound management, cell growth, skin treatment, controlled delivery, oral administration food packaging/ethene adsorption	Yang et al., 2021 [95], Kim et al., 2019 [96], Rizzo et al., 2022 [97], Saad et al., 2021 [98] Wang et al., 2019 [99], Huang et al., 2018 [101], Hassan et al., 2022, Dellali et al., 2021 [103], Xu et al., 2019 [100], Gunathilake et al., 2017 [102] Miyajima et al., 2021 [109]
Films	Blending, co-dissolution	Food packaging/extending shelf life Wound management	Lin et al., 2012 [111], Zhou et al., 2015 [112], Indumathi et al., 2019 [105], Ponnusamy et al., 2022 [106], Zhou et al., 2021 [107], Ghosh et al., 2019 [108] Yang et al., 2019 [113]
Thin Films and Coatings	Adsorption/impregnation/cross-linking of chitosan onto various cellulose substrates: Cellulose fibres (viscose, cotton) coating of cellulose fibres with chitosan nanoparticles, cellulose membranes, and bacterial cellulose	Medical/hygiene/antimicrobial surfaces medical/drug delivery/wound management medical/wound management/antimicrobial medical/wound management/antimicrobial	Cheng et al., 2014 [119], Alonso et al., 2010 [121], Fras Zemljič et al., 2011 [122], Fras Zemljič et al., 2011 [123], Šauperl et al., 2021 [124], Fras Zemljič et al., 2017 [125], Fras zemljič 2014 [126], Ristić et al., 2017 [127] Šauperl et al., 2015 [129], Ristić et al., 2016 [128], Nawalakhe et al., 2013 [162], Li et al., 2018 [164], Zemljič et al., 2020 [163] Cao et al., 2016 [131] Savitskaya et al., 2017 [136], Kingkaew et al., 2014 [137], Khattak et al., 2021 [138], Stanescu et al., 2021 [139], Kai et al., 2020 [140], Hasan et al., 2022 [104], Shen et al., 2021 [141], Xie et al., 2022 [142], Azarmi et al., 2022 [143]
Fibres	Blending/co-dissolution/viscose process/ionic liquids	Antimicrobial surfaces adsorption/cleaning/metal ions adsorption Carbon fibres fabrication	Guan et al., 1998 [90], Hirano et al., 1998 [150], Zhuang 2008 [151], Ma et al., 2012 [152] Zhuang et al., 2021 [153] Zahra et al., 2020 [154]
Nanofibres	Blending/co-dissolution/electrospinning Core–shell electrospinning	Medical/antimicrobial/wound management Wastewater treatment/metal ions adsorption Medical/drug delivery	Du et al., 2008 [155], Park et al., 2011 [159] Phan et al., 2018 [156], Devarayan et al., 2013 [160], Aquino et al., 2018 [161] Kwak et al., 2017 [157], Feng et al., 2019 [158]
